# Review of Chemistry for Students

**Published:** 1851-05

**Authors:** 


Art. VIII.—*Review of Chemistry for Students.* Adapted to the courses as taught in the principal Medical Schools in the United States. By John G. Murphy, M. D. Philadelphia: Lindsay & Blakiston. 1851.

We have been somewhat surprised at the laudatory notices of this work which have appeared in some of the Medical Journals. We cannot even surmise a necessity for the publication, after the recent editions of Bowman and of Fownes, unless it is that those very valuable works are not adapted to the courses as taught in the principal medical schools of the United States, and that this of Dr. Murphy's had the eminent advantage of being thus adapted. But we must say, if Dr. Murphy's work possesses this advantage, its adaptation belongs to a class of medical schools with which we have not the honor of an acquaintance.

The author of this Review of Chemistry certainly has not a very felicitous style for imparting information. He is often very inaccurate in his statements, and when he is not, he frequently hides his meaning in a labyrinth of words that makes the reader oblivious of the intentions of the writer.

We are sorry to speak thus of a book whose purpose is to lighten one of the labors of the medical student, and especially a book in an important branch of medical science that is too much slighted by medical students. Everything that tends to lighten the labor of acquiring chemical science fully, everything that is adapted to the important end of attracting students to the study of chemistry, shall command our approbation and support. We should rejoice to see that important science a much greater favorite than it is with medical students, and we have never been able to see why it is not. To us it is one of the most fascinating of all the departments of medicine, and its claims upon the study of the candidate for the honors of medical science, are in the highest degree imperative.

The work of Dr. Murphy will not greatly advance the interests of chemistry among students of medicine, and will not create a *furor* in its favor anywhere. We regret this for many reasons, one of which is that the medical profession is indebted to Lindsay & Blakiston for many valuable medical books, which have done good service for medical science, and we are sorry to see them send forth such a book as this.

**These various works may be found at the bookstore of Beckwith & Morton. B.**

